# The Potential Role of Nitric Oxide as a Therapeutic Agent against SARS-CoV-2 Infection

**DOI:** 10.3390/ijms242417162

**Published:** 2023-12-05

**Authors:** Shuqi Xiao, Zhiming Yuan, Yi Huang

**Affiliations:** National Biosafety Laboratory, Chinese Academy of Sciences, Wuhan 430020, China

**Keywords:** SARS-CoV-2 infection, COVID-19, NO, antiviral, anti-inflammation, NO therapy

## Abstract

The global coronavirus disease 2019 (COVID-19) pandemic caused by the severe acute respiratory syndrome coronavirus 2 (SARS-CoV-2) has become the greatest worldwide public health threat of this century, which may predispose multi-organ failure (especially the lung) and death despite numerous mild and moderate symptoms. Recent studies have unraveled the molecular and clinical characteristics of the infectivity, pathogenicity, and immune evasion of SARS-CoV-2 and thus improved the development of many different therapeutic strategies to combat COVID-19, including treatment and prevention. Previous studies have indicated that nitric oxide (NO) is an antimicrobial and anti-inflammatory molecule with key roles in pulmonary vascular function in the context of viral infections and other pulmonary disease states. This review summarized the recent advances of the pathogenesis of SARS-CoV-2, and accordingly elaborated on the potential application of NO in the management of patients with COVID-19 through antiviral activities and anti-inflammatory properties, which mitigate the propagation of this disease. Although there are some limits of NO in the treatment of COVID-19, it might be a worthy candidate in the multiple stages of COVID-19 prevention or therapy.

## 1. Introduction

The novel coronavirus disease (also known as COVID-19) caused by the severe acute respiratory syndrome coronavirus 2 (SARS-CoV-2) has been swept across the world and emerged as a major health concern [1] in the recent three years. According to the World Health Organization (WHO), 769,806,130 confirmed cases of COVID-19 have been reported, including 6,955,497 deaths [2]. The fatality rate has varied significantly by region and age groups. Despite commencing vaccination with initially satisfactory efficacy, the SARS-CoV-2 infection has been going on because of many emergences of variants strains, though it has been declared to be a non-concerning disease now. For example, the Omicron variants have caused global concern owing to their great increased transmissibility and immune evasion capability despite its lesser pathogenicity. Therefore, confirmatory polymerase chain reaction tests to identify the SARS-CoV-2 infection and basic preventive measures, such as social distancing and wearing a mask, remain important precautions against COVID-19 [3]. The emergence of the Omicron variants has highlighted the need for more alternative therapies with various modes to reduce the impact of mutated strains such as inhibiting SARS-CoV-2 entry/fusion, RNA replication and protease inhibitors, vaccines and drug-free therapies like plasma therapy, etc., and proposed lifestyle factors (such as nitrate-rich and other natural product diets and exercise) as preventive strategies [4,5].

Nitric oxide (NO) is a key player in both the cardiopulmonary and immune systems, which has already been reported as a worthy candidate for use in the treatment of human coronavirus infections, including COVID-19, because of its antivirus activity and its beneficial effects in the treatment of clinical complications in patients. In fact, inhaled nitric oxide (iNO), as a potent vasodilator, was approved to improve oxygenation in term and near-term neonates, and has been used in clinical settings. Along with its putative antiviral affect, iNO can reduce inflammatory cell-mediated lung injury by inhibiting neutrophil activation, lowering pulmonary vascular resistance, and decreasing edema in the alveolar spaces, thus collectively enhancing ventilation/perfusion matching [6]. However, not enough data from randomized controlled trials are available evaluating the efficacy and safety of NO in COVID-19 presently, and some published data including case reports, cohort studies, retrospective investigations, and in vitro experiments showed conflicting results [7,8]. Some questions remain around factors which may influence whether NO is indeed efficacious or not. This review aims to provide an in-depth discussion on the potential role of NO application in the prevention and therapy of the SARS-CoV-2 infection based on the summary of the mechanism of the SARS-CoV-2 infection and its pathogenicity.

## 2. SARS-CoV-2 and COVID-19

SARS-CoV-2 is a member of the coronaviruses (CoV) family, which are enveloped and single-stranded positive-sense RNA viruses with (typically) a genome of ~30 kilobase (kb). Coronaviruses also have been named after the protruding coronary spikes on the virus’s surface [9]. Similar with other β-coronaviruses genome organization, SARS-CoV-2 consists of un-translated regions(UTRs) at both 5′ and 3′ end regions and fourteen functional open reading frames (ORFs) that encode for different structural proteins, non-structural proteins (nsps), and accessory proteins [10,11]. There are four structural proteins: the spike (S) protein encoded by the S gene is the site on the virus’s surface responsible for binding to the host receptor; the M protein encoded by the M gene shapes the virions and directs envelop formation and provides the matrix for nucleocapsid attaching and budding; the E protein encoded by the E gene is involved in the virus’s assembly and release, contributing to the pathogenesis; and the N gene encodes the N protein, which binds to the RNA genome to maintain the virus’s stability. The ORF1a and ORF1b encode sixteen highly conserved nuclear shuttle proteins (nsp1-nsp16) that are essential for viral replication and transcription processes. Nine accessory proteins provide a selective advantage in the infected host [12,13,14].

Apart from SARS-CoV-2, the other two coronaviruses were found to transmit to human populations, trigger acute respiratory syndromes in humans, and even cause severe outbreak clusters after overcoming the species barrier over the recent two decades, namely the severe acute respiratory syndrome (SARS-CoV-1) in 2002 and the Middle East respiratory syndrome (MERS-CoV) in 2012 [15,16]. SARS-CoV-2 shares 80% and 50% similarity with SARS-CoV-1 and MERS-CoV, respectively [17]. All of them belong to the β-coronavirus genera in the coronaviruses family which infects the respiratory tract, causing atypical pneumonia, and also affects the function of other organs like the liver, heart, kidney, gastrointestinal system, and central nervous system [18].

COVID-19 is either asymptomatic or mild, with the most common symptoms being fever, headache, dry cough, shortness of breath, and myalgia in about 80–90% of cases, and only around 10% of the infected patients have severe infection with dyspnea, hypoxemia, and extensive radiological involvement of the lung parenchyma. In some critical cases (less than 5%), this virus is likely to cause acute lung injury (ALI), acute respiratory distress syndrome (ARDS), sepsis, and subsequent multi-organ failure leading to respiratory failure and eventually death, which are very similar to the pathological features of SARS and MERS [19,20]. Thus, the symptoms vary from individual to individual, ranging from an asymptomatic infection to severe respiratory failure. Gastrointestinal disorders, such as diarrhea, nausea, and vomiting, are reported to a lesser extent. Some patients have also experienced loss of smell and nasal obstruction. This indicated a potential neurotropism of SASR-CoV-2 that may invade the central nervous system [21]. The individuals with pre-existing comorbidities like obesity, hypertension, diabetes, chronic obstructive pulmonary disease (COPD), cardiovascular disease, cerebrovascular disease, and autoimmune disease or immunosuppressed condition are at a much higher risk of severe COVID-19 disease [22].

**Figure 1:**
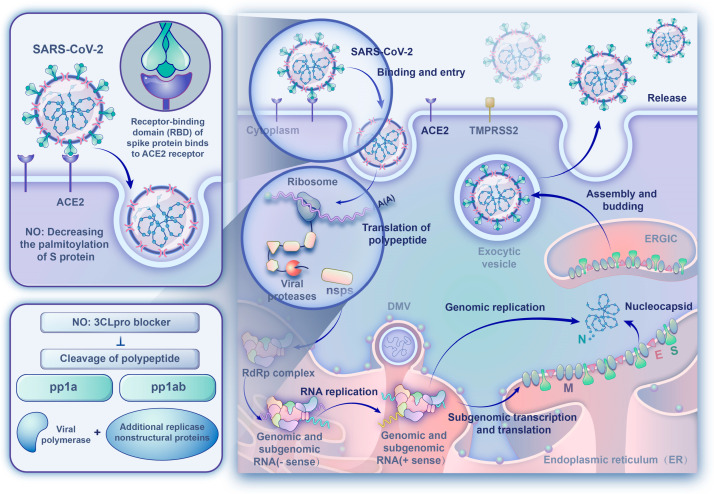
The severe acute respiratory syndrome coronavirus 2 (SARS-CoV-2) lifecycle and the antiviral effect of NO. The details are described in the text.

Although SARS-CoV-2 mainly infects bronchial ciliated epithelium and pulmonary type II cells initially, electron imaging has detected leftover virus particles in endothelial cells. SARS-CoV-2’s entry into host cells is mediated by binding to the host cellular receptor, angiotensin-converting enzyme 2 (ACE2), which is located on the host cell surface of the target organs, with a higher affinity than with the one measured with SARS-CoV-1. ACE2s are highly expressed in type II alveoli epithelial cells, which serve as the primary targets for viral attacks [23]. Studies identified that the virus significantly impacts other organs with the development of myocarditis, gastrointestinal disturbances, renal ailments, and irregular blood pressure, and the presence of ACE2 on the epithelial and endothelial lining of the liver, heart, kidney, pancreas, gastrointestinal tract, genital organs, thyroid, blood vessels, and so on is considered partly responsible for this [24]. In the case of SARS-CoV-2, S protein, which is required for viral entry, has two regions, S1 and S2. S1 has a receptor-binding domain (RBD) that mediates direct contact with ACE2 to form the S protein RBD-ACE2 complex, whereas S2 is involved in subsequent membrane fusion [25]. SARS-CoV-2 contains two cleavage sites at the boundary of the S1/S2 subunits, which is very different from other β coronaviruses. Following receptor binding, the S protein is proteolytically cleaved at the S1/S2 and S2 sites by transmembrane protease serine 2 (TMPRSS2) [26,27]. Then, the followed conformational change may cause a close apposition of S protein to the cellular membrane [28]. And, the membrane fusion and viral entry are facilitated due to increasing lipophilicity when the cysteine residues of S protein are palmitoylated (Figure 1).

After SARS-CoV-2 enters the target cells, the virus is disassembled to release viral RNA into the cytoplasm for translation of non-structural proteins and structural proteins and replication of genome. The translated replicase components rearrange the endoplasmic reticulum (ER) into double-membrane vesicles (DMVs) that facilitate viral replication of genomic and subgenomic RNAs. The latter are translated into accessory and viral structural proteins to facilitate virus particle formation. The virus particles germinated in the endoplasmic reticulum–Golgi intermediate compartment (ERGIC) were exocytosed into the extracellular compartment for the propagation of the infection in other target cells [29]. Similar to SARS-CoV-1, the SARS-CoV-2 3-chymotrypsin-like (3CL) cysteine protease, as an essential non-structural protein for the life cycle of the virus, cleaves viral polyproteins into effect proteins, and could be an attractive antiviral target because the observed reduction in the protease activity was consistent with S-nitrosylation of enzyme active site cysteine and consequent reduction in viral replication [30,31] (Figure 1).

**Figure 2:**
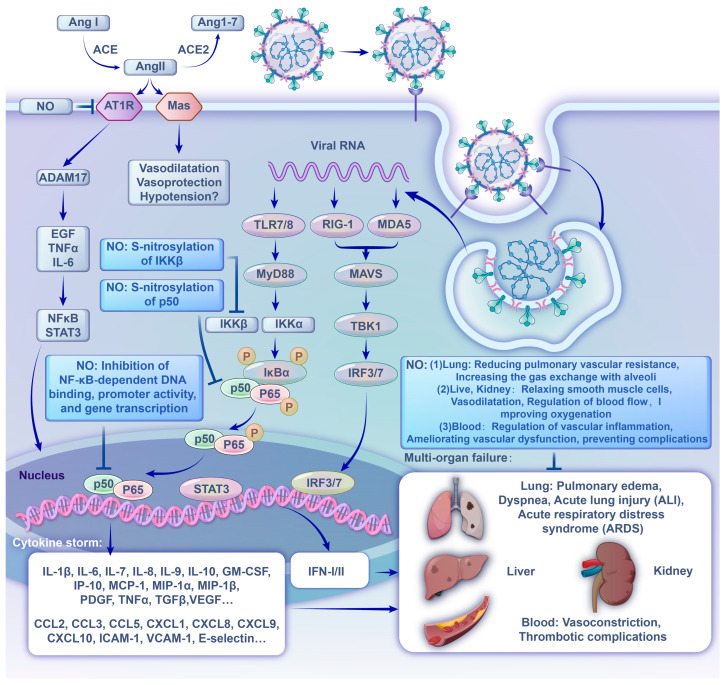
Brief overview of SARS-CoV-2 recognition by innate immune system and subsequent inflammatory process and the pathogenesis and clinical manifestation of COVID-19, and the effect of NO in these processes.

In parallel with the ongoing viral replication, the viral RNA genome release into the cytoplasm is detected by intracellular pattern recognition receptors (PRRs), innate immune sensors such as the endosomal Toll-like receptor, and cytosolic retinoic acid-inducible gene I-like receptors. Following PRR activation, molecular signaling cascades culminate in the activation of downstream transcription factors, such as nuclear factor-κB (NF-κB), to produce numerous pro-inflammatory cytokines (including interferon (IFN)-α, IFN-γ, interleukin (IL)-1β, IL-6, IL-12, IL-18, IL-33, tumor necrosis factor (TNF)-α, and transforming growth factor (TGF)-β) and chemokines (such as chemokine ligand (CCL) 2, CCL3, CCL5, CXC chemokine ligand (CXCL) 8, CXCL9, and CXCL10) in the form of a “cytokine storm” [29,32]. Multiple clinical symptoms are strongly connected with the release of these cytokines. For example, they are connected with IFN-γ production, which results in headache, chills, dizziness and fever, and vascular leak syndrome caused by the production of IL-6, which activates the coagulation and complement pathways [33,34]. These inflammatory mediators further damage the lining of epithelial cells and reach the bloodstream where they cause ARDS and multiple organ failure, and finally lead to death in severe cases of the SARS-CoV-2 infection. A similar phenomenon occurs in the SARS-CoV and MERS-CoV infections [19,35,36]. Animal models have also illustrated apoptosis of the epithelium and endothelium, and subsequent vascular permeability and abnormal T-cell and macrophage responses induced with ALI and ARDS. A hyper-inflammatory response and compromised vasculature are increasingly shown to spur multisystem dysfunction [19]. Therapies that can modulate the inflammatory cascade or cytokine storm may prevent the rapid progression to the ARDS and systemic organ failure, which are driving factors for the incidence of mortality in patients with COVID-19 (Figure 2).

Another pathway pertaining to the pathogenesis of COVID-19 is the ACE2-angiotensin 1-7 (Ang 1-7) axis. It is known that ACE converts angiotensin I (Ang I) into the pro-inflammatory peptide angiotensin II (Ang II), and ACE2 metabolizes Ang II to produce Ang 1-7. The SARS-CoV-2 infection downregulates ACE2 expression by internalizing it with viral particles from the host cell surface and fails to catalyze the conversion of Ang II to Ang 1-7, resulting in Ang II accumulation. A high concentration of Ang II can cause increased inflammatory responses and reactive oxygen species (ROS) [37,38]. A large number of activated pro-inflammatory cytokines and chemokines were found in the serum of patients with severe COVID-19, including the membrane forms of epidermal growth factor (EGF) family members, IL-6 receptor, and TNF-α, and developed into a strong cytokine storm [39]. When high inflammation persists for a long time, it damages many tissues and organs and contributes to an imbalance of ROS, which leads to vasoconstriction. As a feedback loop, when excessive ROS are present, endothelial injury is aggravated by cell apoptosis, triggering of transcriptional factors (such as NF-κB and signal transducer and activator of transcription-3 (STAT3)), and overexpression of inflammatory cytokines and adhesion molecules (ICAM-1, VCAM-1, E-selectin, etc.) [40]. Furthermore, the SARS-CoV-2 infection may promote a persistent state of inflammation and endothelial dysfunction long after the viral particles have been cleared from the body by inducing oxidative stress [41] (Figure 2).

## 3. Role of NO in SARS-CoV-2 Infection

NO is involved in a wide range of physiological processes by reacting with various reagents, and it has been established that NO has a role in the pathogenesis of many viral infection as well as direct or indirect antiviral activity, such as directly inactivating viral particles, inhibiting their replication, or modulating the host immune response. In COVID-19, NO was also reported to function from the following potential mechanisms, including antiviral effector, anti-inflammation, anticoagulation, vasodilation, etc.

### 3.1. Antiviral Effect

From the early days, it was warranted that NO has a rather broad spectrum of antiviral effects and inhibits viral replication, including ectromelia virus, vaccinia, vesicular stomatitis virus, adenovirus, murine CMV (MCMV), murine retrovirus, rhinovirus, herpes simplex-1 viruses, HIV, hantavirus, influenza, Japanese encephalitis virus, and (most importantly) coronavirus [42,43,44,45,46,47,48,49,50,51,52,53,54,55,56,57,58,59,60,61,62,63]. More notably, NO has been shown to impair SARS-CoV and SARS-CoV-2 replication in light of the COVID-19 pandemic because of their similar infection processes [30,31,64,65]. Two NO-mediated antiviral mechanisms were proposed, and were later experimentally verified (Figure 1), as follows:

(1) NO decreased the palmitoylation level of S protein, thereby interfering with binding to the target receptor on the host cell. Three different studies demonstrated the potential of NO compounds in the inhibition of SARS-CoV replication in a concentration-dependent manner [30,64,65], and the effect of NO on S protein was also investigated. The results showed that the NO donor-S-nitroso-N-acetylpenicillamine (SNAP) treatment significantly reduced the number of palmitoylated S protein, and the intercellular fusion was significantly decreased. Also, the entry efficiency of the pseudo-type virus was significantly lower after SNAP treatment, and the virus infection rate decreased by about 70% [30].
(2) NO affected replication-related cysteine proteases which directly inhibited viral RNA replication. Similar to the Coxsackievirus 3C cysteine protease, SARS-CoV-2 3CL cysteine protease may be a potential target for S-nitrosation, causing a suppression of the protease activity and a resultant decrease in viral replication [56]. The in vitro study by Akaberi et al. showed that SARS-CoV-2 3CL recombinant protease was covalently inhibited by SNAP through the transfer of nitrosonium ions (NO^+^s) to the protease cysteine residue, and the observed reduction in SARS-CoV-2 protease activity was consistent with S-nitrosylation of the enzyme active site cysteine. Although the viral replication was not completely abolished, SNAP delayed or completely prevented the development of the viral cytopathic effect in treated cells [31]. In addition, the analysis of proteolytic degradation of the viral polypeptide showed that the content of the nucleocapsid N protein was drastically decreased in the presence of SNAP and that high-molar-mass (non-processed) polypeptide content was increased [30,65].

In addition, endogenous NO also enhances ciliary beat frequency of the nasal respiratory epithelium, facilitating mucociliary clearance of pathogens [66]. Despite the above, the mechanism of NO in the SARS-CoV-2 infection requires further investigation. NO, together with other clinically antiviral drugs, is recommended as an effective strategy for the treatment of COVID-19.

### 3.2. Effect on Inflammation

Inflammation is a critical defensive mechanism for inactivating pathogens, removing irritants, and paving the way for tissue repairs. However, excessive inflammation causes injury. Studies have shown that NO, as a ubiquitous signaling molecule, plays a role in almost every stage of inflammation [67] (Figure 2).

For example, NO suppresses the production of a large number of cytokines in lymphocytes, eosinophils, monocytes, and other immune cells, including key cytokines in the inflammatory response [54,68,69]. The different and inappropriate inflammatory response associated with the SARS-CoV-2 infection in the context of the COVID-19 illness was described above (SARS-CoV-2 and COVID-19 section). Moreover, multiple reports indicated that NF-κB is involved in the upregulation of inflammatory responses in patients with the SARS-CoV-2 infection as a potential main regulator of this process [70]. Regulation of NF-κB activation by NO has been well investigated for its involvement in various physiological and pathological conditions [71]. The most common form of active NF-κB is a heterodimer consisting of the protein subunits p50 and p65. After the IκB-kinase (IKK) complex phosphorylates the cytoplasmic inhibitor factor IκB, which normally sequesters NF-κB in an inactive form in the cytosol, NF-κB is translocated into the nucleus and induces a plethora of pro-inflammatory gene expressions. Here, NO represses IκB-kinase through S-nitrosylation and S-nitrosylation of IKKβ at Cys179, and p50 at Cys62 inhibits NF-κB-dependent DNA binding, promoter activity, and gene transcription, leading to the subsequent inflammatory response [72]. In this context, the NO-mediated inhibition of the NF-κB pathway might be a potential therapeutic option for COVID-19. Furthermore, NO suppresses immune cell growth during inflammatory responses [73]. As a result, extreme inflammatory effects, such as the cytokine storm, are reduced, and unrestrained physical injury is avoided by NO [74].

An additional factor that contributes to the excessive inflammatory responses is relevant to ACE2. ACE and ACE2 serve opposing physiological functions. After ACE cleaves Ang I to Ang II, Ang II binds its receptor to constrict blood vessels. In addition, ACE inhibits NO production, promoting ROS and inflammation. An excess of ROS damages endothelial dysfunction, permeable vessels, and lipid membrane peroxidation [75]. On the contrary, ACE2 inactivates Ang II and generates Ang 1-7, which promote endothelial production of NO, as both potent vasodilators and inhibitors of ACE. The accumulation of Ang II caused by the downregulation of ACE2 expression in the SARS-CoV-2 infection can induce vasoconstriction and act as a pro-inflammatory cytokine via AT1R. The AngII-AT1R axis induces inflammatory cytokines, including TNFα and IL-6-soluble (s)IL-6R, via activating disintegrin and metalloprotease 17 (ADAM17), followed by the activation of the IL-6 amplifier (IL-6 AMP), which describes enhanced NF-κB activation machinery via the coactivation of NF-κB and the signal transducer and activator of transcription-3 (STAT3) [39]. This in vitro study showed that the NO donor, sodium nitroprusside, dose-dependently inhibited the binding affinity of AT1R with Ang II by S-nitrosylation of AT1R at cysteine 289 [76]. The treatment of rats with oral nitrite increased circulating S-nitrosothiol levels and reduced Ang-II-induced vasoconstriction [77]. These results suggest the potential beneficial effects of NO in deleterious COVID-19 progression associated with Ang II.

Inflammation-induced platelet activation, which can lead to increased coagulation and consequent diseases, can be lessened by NO [78]. NO maintains physiological vascular homeostasis in tissues and protects blood vessels from damage with platelets and circulating cells, and the decrease in endothelial NO production is a sign of endothelial dysfunction and thrombotic events [79]. The decreased or ceased release of NO following endothelial cell dysfunction leads to the accumulation of free Ca^2+^ in vascular smooth muscle cells, continuous vasoconstriction, and subsequently a blood hypercoagulable state. When blood vessels are damaged, platelets quickly gather to the injured site to form platelet clots and a complex with plasma factor VIIa, whose subsequent interaction with extravascular tissue factor initiates the action of thrombin (via conversion of inactive protease factor X into the active protease factor Xa). Thrombin then converts soluble fibrin into insoluble fibrin, which makes the platelet clot entangled with blood cells to form a thrombus. At the same time, platelets contain vascular growth factors and release a variety of pro-inflammatory mediators. Recent studies showed that a hypercoagulable state and the thrombotic complications caused by excessive platelet activation were both important pathological inflammatory events in severe COVID-19 patients [80]. Cheng et al. also recently reported evidence that enhanced clotting and sluggish blood flow result in systemic hypoxia in oxygen-sensitive organs, such as the kidneys [81].

### 3.3. Effects on Vasodilation

NO can serve as an effective vasodilator regulator. It effectively relaxes smooth muscle cells and dilates blood vessels to improve oxygenation and reduce pulmonary vascular resistance and promoter oxygen inhalation, thus increasing the blood flow of capillaries, increasing the exchange gas with alveoli, and accelerating oxygen circulation in the body, which may improve respiratory symptoms. 

In detail, NO reacts with oxygen to form nitrogen dioxide and nitrite, resulting in pulmonary vasodilatation. Further, nitrosylation of the cysteine residue of the haemoglobin β subunit leads to the formation of a stable derivative that retains vasodilatory properties which can increase blood flow and oxygen delivery to the system’s vasculature [82]. NO regulates the vascular tone via the cyclic guanosine monophosphate (cGMP)-dependent mechanism. It binds to soluble guanylate cyclase (sGC) and activates it, resulting in the production of intracellular cyclic guanosine monophosphate (cGMP). cGMP reduces the intracellular Ca^2+^ concentration and relaxes smooth muscle cells. The reduction in calcium reduces the ability of myosin light-chain kinase (MLCK) to phosphorylate the myosin molecule, preventing cross-bridging and thus enhancing the relaxation of smooth muscle cells and promoting blood flow. It also activates potassium channels, leading to hyperpolarization and relaxation [83]. In addition, NO is also involved in the metabolic pathways of nitrosothiol (RSNO), which has a strong bronchiectasis effect independent of the cGMP pathway, which effectively improves airway tension and increases oxygen intake [84].

Overall, NO is involved in vascular signaling and the regulation of blood flow. Vasculature depleted of NO suffers from persistent inflammation and a decreased delivery of oxygen and removal of toxic byproducts through stagnant blood flow into and out of hypoxic tissues [85,86,87]. In addition, NO therapy may play key roles in the regulation of vascular inflammation, ameliorating vascular dysfunction and preventing complications, such as tissue edema and respiratory failure caused by vascular leakage [87]. 

## 4. Application of NO in Clinical Treatment of COVID-19

Based on the aforementioned antiviral, anti-inflammatory, and anti-thrombotic effects, NO, also as a potent and selective pulmonary vasodilator, has been appraised as an attractive agent that may be beneficial to COVID-19 patients’ therapy, with or without ARDS [6,7]. Further, NO inhalation therapy showed promising potency in the 2003 SARS outbreak [88]. Many case series, cohort studies, retrospective investigations, and clinical trials that investigated different strategies of NO administration under various conditions were conducted and analyzed to discuss the use of exogenous NO therapy among patients with COVID-19 (Table 1).

Many clinical observations and studies have demonstrated that iNO treatment produced an acute improvement in the systemic oxygenation process in hypoxemic patients and prevented the progression of hypoxemic respiratory failure [103,110]. High-dose NO (160–200 ppm for 30 min) was safely administered to pregnant females with severe COVID-19 pneumonia and was associated with improved oxygenation, respiratory rate, and cardiopulmonary system function and a decrease in systemic inflammation [93]. Another strategy for iNO administration in COVID-19 is the administration of a long-term, constant NO insufflation at low doses, which may increase antiviral activity (dose and time-dependent) and reduce the severity of the disease and time to recovery in patients with COVID-19 [122,123]. Intriguingly, a randomized clinical trial using iNO among healthcare workers was conducted to prevent them from being infected with the SARS-CoV-2 during their work (Table 1). The use of iNO in conjunction with pharmaceutical vasodilators, such as almitrine and prostaglandin, has also shown a positive clinical value as a rescue therapy to enhance oxygen levels in patients with COVID-19 [98,115,119]. However, several trials showed that iNO treatment did not improve oxygenation in patients with COVID-19 and refractory hypoxemia [100,101]. The varied timing of administration and dose may partly account for the conflicting results in these studies. Additionally, the level of cellular debris and tissue damage during different COVID-19 stages may be too overwhelming to enable iNO to be clinically beneficial. The comprehensive literature search combined with systematic analysis highlighted the clinical value of iNO, which suggests a relatively high benefit in terms of improved arterial oxygenation, and recommended the integration of iNO in the routine clinical management plan of patients with COVID-19 [7].

In addition to iNO, donor compounds which boost NO production may also provide protection against viral proliferation and the many adverse pulmonary and vascular consequences in patients with COVID-19. However, no trials with donor compounds or natural products are currently underway, and their efficacy should be explored for early interventions regarding COVID-19.

## 5. Discussion

The endothelial NO production or bioavailability drops off in elderly patients or those suffering from hypertension, diabetes, obesity, chronic obstructive pulmonary disease (COPD), autoimmune disorders, and cardiovascular diseases [124,125,126,127]. Older COVID-19 patients with preexisting endothelial dysfunction may exhibit an increased vulnerability to COVID-19, and their condition is likely to worsen (physically) in comparison to their younger and healthier counterparts [128,129,130,131]. Exogenous NO therapy at the optimal stage of infection for these vulnerable populations may be an accessible, compelling option, which can reduce viral load, prevent the chain of events that rapidly destabilizes patients to ARDS, and promote clinical recovery. In addition, supplementation with NO donors may hold promise for boosting resistance to respiratory infections, including COVID-19, in older individuals, despite its benefits for cardiovascular health [132]. However, varying levels of efficacy and safety were shown depending on the protocol used and the complex physiological pathways involved in NO production and regulation. 

## 6. Conclusions

The spread of the COVID-19 infection across the world has become the largest public health challenge of recent years, and the disease’s complications associated with endothelial dysfunction are multifactorial. Furthermore, new pandemics are likely to await humanity in the future, along with new emerging respiratory viruses with a pandemic potential appearing in the world. All of these have highlighted the need for developing new strategies to face the uncertainty in the prevention and therapy of these viruses based on the multitude of pathogenic mechanisms, especially for vulnerable populations. This review elaborated on the pathogenesis of the SARS-CoV-2 infection and the potential role of NO therapy in patients with COVID-19 through antiviral activities and anti-inflammatory properties in relieving disease-related symptoms. Exogenous NO may be a safe and prospective approach for the prevention and treatment of patients with COVID-19, including prevention of infection, intervention of mild patients, alternative rescue treatment of moderate and severe patients, and adjunct therapy. However, more studies focusing on the safety and efficacy of NO therapy regimens in patients with endothelial function failure associated with COVID-19 or other respiratory viral infections are required. Moreover, the development of personalized therapeutic protocols for better prognoses of severe patients is also extremely important. Clinicians and researchers should work together to define the potential role of NO against the SARS-CoV-2 infection and its molecular mechanisms for the wider introduction of NO therapy into clinical practice.

## Figures and Tables

**Table 1:** Summary of studies on NO in the treatment of COVID-19.

	Experimental Design	Dose	Duration	Sample Size	Finding	Reference
1	Double-blind randomized study	120–140 μL of solution/spray	24 h/72 h	80	NONS accelerated the reduction in SARS-CoV-2 RNA load versus a control with saline spray.	[89]
2	Retrospective study	10–20 ppm (parts per million)	5–13 days	20	The use of NO or almitrine bismesylate or both did not improve oxygenation in moderate to severe COVID-19 ARDS, but the result remains to be confirmed by a study on a larger cohort of patients.	[90]
3	Retrospective observational study	20 ppm	15–30 min	7	iNO provided immediate help and delayed respiratory deterioration in COVID-19-induced moderate to severe ARDS.	[91]
4	Multicenter, retrospective cohort study	20–40.0 ppm	24 h–30 days	1598	In critically ill COVID-19 patients with moderate-to-severe ARDS, iNO rescue therapy is related to improved oxygenation parameters but no mortality benefits.	[92]
5	Retrospective observational study	160–200 ppm	30 min–1 h	6	High-dose iNO was well tolerated and improved oxygenation and respiratory rate for pregnant patients with severe or critical COVID-19.	[93]
6	Retrospective cohort study	200 ppm, 30 min	twice daily, 23–26 days	71	iNO treatment improves respiratory function and outcomes among pregnant patients hospitalized with severe COVID-19 pneumonia.	[94]
7	Multicentric cohort study	5–30 ppm	2–11 days	164	iNO was related to severe AKI and RRT in critically ill patients with COVID-19.	[95]
8	Retrospective observational study	10–20 ppm	24–30 days	5	iNO treatment was beneficial in reducing and stabilizing the PASP and reduced the risk of right heart failure in patients with COVID-19 with pulmonary hypertension.	[96]
9	Observational study	40 ppm	24 h	12	COVID-19-related severe ARDS iNO administrated as rescue therapy cannot ameliorate oxygenation nor pulmonary hypertension.	[97]
10	Single-center retrospective observational study	10 ppm	2–5 days	32	Almitrine often used in combination with iNO improves oxygenation in patients with SARS-CoV-2-induced ARDS without side effects, and in the case of life-threatening refractory hypoxemia, almitrine alone or in combination with iNO could be a good time saver.	[98]
11	Single-center prospective study	10 ppm	15–30 min	34	iNO improves PaO_2_/FiO_2_ ventilation/perfusion in the majority of patients with COVID-19 and severe pneumonia.	[99]
12	Observational study	20 ppm	30 min	10	iNO relieves hypoxemia in mechanically ventilated COVID-19 patients.	[100]
13	Single-center, observational study	20–30 ppm	15–30 min	72	iNO induced an improvement in oxygenation and cardiac output.	[101]
14	Retrospective cross-sectional study	20–40 ppm	2–7 days	34	iNO is an auxiliary therapy that can increase the PaO_2_/FiO_2_ ratio in SARS-COV-2 mechanical ventilated critically ill patients without major side effects.	[102]
15	Single-center observational study	30 ppm	2.1 days	39	iNO therapy prevents the progression of hypoxic respiratory failure in patients with COVID-19.	[103]
16	Multicenter cohort study	20–40 ppm	44–135 h	272	iNO therapy improved oxygenation in spontaneously breathing patients with COVID-19.	[104]
17	Retrospective cohort study	10–40 ppm	6 days	84	iNOs are not associated with improved gas exchange in mechanically ventilated patients with COVID-19.	[105]
18	Single-center retrospective study	20–30 ppm	18–72 h	38	A group of patients showed a significant improvement with inhaled nitric oxide. The administration of inhaled nitric oxide may be considered in patients with severe respiratory failure secondary to COVID-19.	[106]
19	Single-center retrospective case–control study	10–20 ppm	24 h	154	iNO improves oxygenation in COVID-19-related acute respiratory distress syndrome.	[107]
20	Prospective observational study	20 ppm	1 h	22	iNO and prone positioning improved systemic and cerebral oxygenation.	[108]
21	Retrospective study	20–40 ppm	2–72 h	59	Both iNO and prostaglandins can be used in patients with COVID-19 with severe refractory hypoxaemia.	[109]
22	Multicenter interventional study	160 ppm, 30 min	twice daily, 4–8 days	29	A high dose of iNO is helpful in spontaneously breathing patients with COVID-19.	[110]
23	Observational study	20–30 ppm	24 h	1	Continuous iNO-enriched ventilation was effective in a patient with COVID-19 on veno-venous ECMO.	[111]
24	Retrospective observational study	20 ppm	5 days	35	iNO can be used in patients with COVID-19 with refractory hypoxaemia.	[112]
25	Multicenter randomized study	140–180 ppm for 30 min	2 sessions every day for 14 days	1260	A protocol was developed to treat patients with COVID-19 with NO in RCTs.	[113]
26	Retrospective observational study	9–40 ppm, ≥24 h	3–9 days	37	iNO is helpful for the treatment of mild-to-moderate ARDS in patients with COVID-19.	[114]
27	Monocentric prospective study	10 ppm, 30 min	5–15 days	10	The iNO-almitrine combination is effective in oxygenation improvement.	[115]
28	Single-center, retrospective cohort study	20–80 ppm	2–36 h	122	Prone positioning is helpful in oxygenation among patients treated with iNO.	[116]
29	Randomized study	250 ppm, 30 min	28 days	47	Single high-dose iNO is effective in patients with acute respiratory symptoms.	[117]
30	Multicenter randomized study	0.45 mL/dose	six time daily, 7 days	306	NONS accelerates nasal virus clearance in patients with COVID-19.	[118]
31	Single-center, open- label, observational study	10 ppm, 30 min	3–19 days	12	The combination of iNO and almitrine improved short-term oxygenation in patients with COVID-19.	[119]
32	Single-center, retrospective observational study	160 ppm, 30 min	twice daily, 1–9 days	5	Gaseous NO is a useful adjuvant rescue therapy for patients with COVID-19.	[120]
33	Single-center, randomized study	160 ppm, 15 min	twice daily, 14 days	470	Inhalation of NO can prevent COVID-19 among healthcare workers.	[121]

NONS: NO nasal spray; ppm: parts per million; ARDS: acute respiratory distress syndrome; iNO: inhaled NO; AKI: acute kidney injury; RRT: renal replacement therapy; PASP: pulmonary artery systolic pressure; PaO_2_/FiO_2_: the ratio of arterial oxygen partial pressure to fractional inspired oxygen; ECMO: extracorporeal membrane oxygenation; RCT: randomized controlled trials.

## Data Availability

Not applicable.
